# Bovine Tuberculosis*Read before the Haverhill Med. Club, April, 1893.

**Published:** 1893-10

**Authors:** John M. Parker


### BOVINE TUBERCULOSIS.*

By John M. Parker.

I am pleased to have this opportunity of bringing the subject of Bovine Tuberculosis before you, not because I have anything particularly new or original to present to you, but because it is a matter of special importance and because I wish to call your attention especially to the sanitary and hygienic aspects of the subject, which have been too much neglected.

As you are aware, all domestic animals are more or less subject to Tuberculosis. Dairy cattle however, in consequence of their mode of life and because of the heavy drain on their system through excessive breeding and milking, are more predisposed than any other of the domestic animals.

* Read before the Haverhill Med. Club, April, 1893.

Of the different breeds of cattle, the delicate, highly bred Jersey is probably the most susceptible, while the hardy native breeds are most exempt.

The early physical diagnosis of Bovine Tuberculosis is difficult; the symptoms are very meagre, and in fact in some cases, even when the disease is well advanced, there is seemingly little alteration in the health of the animal.

For example, in that form of Tuberculosis known as "Perlsucht" or "Pearl disease" of cattle, where the serous membranes are covered with the peculiar characteristics nodular masses, the animal may be in a seemingly perfectly healthy condition.

At other times the only symptoms observable may be enlargement or tumefaction of the external lymphatic glands, with possibly tubercular nastitis, in which condition there may be a characteristic hard and knotted condition of the udder which is devoid of sensibility and is nonsecretive, or there may be a "diffuse and uniform enlargement and induration" with "functional activity to a greater or less degree."

These symptoms, however, would be sufficient, especially with a previous history of tuberculosis in the herd, to comdemn the animal.

Again there may be indigestion and persistent tympanitis from the enlarged bronchical glands pressing in the æsophagus, and so mechanically preventing regurgitation of food or gas from the stomach.

Persistent œstrum or heat with barrenness, especially when there is a harsh, unthrifty condition of the coat and a general loss of condition, must be looked upon with suspicion.

Tubercular Arthritis is also common in dairy cattle.

The most common form of tuberculosis, however, is where there is an almost characteristic chronic cough; on exertion the breathing becomes hurried and more labored and is usually accompanied or followed by the cough. Auscultation and percussion show a more or less diseased condition of the lungs. There is usually more or less marked tumefaction of one or more of the superficial lymphatic glands. There is often scouring, the buttocks being in a dirty state. The temperature may or may not be altered and the pulse but little affected.

The only positive diagnostic symptom is the finding of the bacilli in either the nasal or vaginal discharge, pharyngeal mucus, or the milk.

The microscopical examination of the nasal discharge, when present, usually yields the best results.

The only other method in use as aids to diagnosis are the inoculation of the suspected animal with Koch's tuberculin and the inoculation of rabbits or guinea pigs with either discharge or milk.

The first of these methods (Koch's tuberculin) has been extensively used and highly recommended by both European and American investigators, for diagnostic purposes, and while it has not yet been recognized as an infallible diagnostic agent, the results have been sufficiently encouraging to warrant further trial and investigation.

The second of these methods, the inoculation of rabbits or guinea pigs with the milk or the discharge from a suspected animal, will probably never be brought into general use by the ordinary practitioner. In a suspicious case, however, it is often of the greatest value as an aid to diagnosis.

*Aetiology.*—As you are aware the immediate cause of Bovine Tuberculosis is the same as in the human family (viz. Koch's bacilli).

The bacilli do not always present the same appearance however, certain "morphological differences are found," and "under different circumstances and within limits the morphology of the tubercle bacilli varies with its environments." Thus tubercle bacilli from the nodulus of "perlsucht" are generally shorter and thicker than those from the human being. Those found in cows' milk again aproach more nearly to the familiar rod shaped bacilli found in the human sputa; but while minute morphological differences can be detected in the tubercle bacilli of different species of animals, the general characters and characteristics of staining and culture are the same in them all.

In considering the aetiology of Bovine Tuberculosis there are two natural groupings into which the methods of infection can be divided.

The first and most important being the introduction of the bacilli through the respiratory tract.

The second, the direct introduction of the germ through congenital or hereditary transmission, or through eating or drinking tuberculous meat or milk.

In considering the first group, it is necessary to take a generally broad view of the subject and include all sources from which the tubercle bacilli can gain access to the atmosphere, whether that source is a consumptive human being or a tubercular cow matters not. *Once they gain entrance to the atmosphere, from whatever source they come, they are equally dangerous.*

The two most important means by which the bacilli can gain admittance to the atmosphere are: First, by the expectorations of consumptives.

Second, by the nasal discharge of diseased animals.

In reviewing the work on the subject of phthisical expectorations, in the Annual of Medical Science for 1890, Whitaker says: "The work of the year has established almost to universal conviction that pulmonary tuberculosis is caused exclusively by inhalation of dried sputum." This, the original postulate of Koch, met its conclusive proof in the studies of Cornet; "it is not," he says, "the breath of the consumptive which is dangerous, but singly and alone the inhalation of the dried sputum which is mixed with the dust of the floor."

"The whole question of infection," he goes on to say, "has in the past year narrowed itself down to infection by sputum and by milk, and this fact may be regarded as the acquisition of the year." (Man. of Med. Sc., P. A 8, 1890.)

We can easily understand therefore how a consumptive person having charge of a dairy herd must become a source of danger to that herd, and might become a source of greater danger to a healthy herd than even the introduction of a tuberculous animal.

An instance was related to me some time ago by one of the State Board of Cattle Commissioners having a direct bearing on this subject. In his capacity of State Inspector, he was called to see a young heifer which was ailing. There was no previous history of disease in the herd; all the animals, so far as could be traced, were perfectly healthy; no new animals had been brought in; the bull used for service was a young, healthy animal, with no trace of disease; and yet unmistakable symptoms of tuberculosis began to develop in this young heifer. (These on post mortem examination proved to be tuberculous.)

Here was something of a puzzle, which was easily explained when it appeared on conversation that one of the attendants was far gone with acute phthisis. And I believe if this matter were carefully investigated, like causes would be found far more common than is generally supposed.

The second source of danger, viz. the nasal discharge of diseased animals, is analogous to the expectorations of human consumptives.

Cattle do not actually expectorate, but we must remember their bodies are not in the upright position of a human being, their heads are held lower and consequently any discharge from the lungs runs freely from the nasal passages; in this way the manger and woodwork become covered with the discharge, which dries, and subsequently particles becoming detached, mix with the dust and dirt, and become a source of danger to the neighboring animals.

The second group of ways, by which the bacilli may gain entrance to the animal body, is by direct infection through eating or drinking tuberculous meat or milk or by direct hereditary transmission.

In the matter of diseased meat, the divergence of opinion on the subject is peculiar.

Bollinger, Kastner, Nocard, and others have come to the conclusion "that the flesh of tuberculous animals is only exceptionally dangerous and even in these exceptional cases it is dangerous only in a slight degree."

Other observers hold the opposite opinion, and at the Congress for the study of Tuberculosis, held in France in 1889, and at the Congress of Hygiene and Demography, held in London in 1890, resolutions were passed recommending absolute seizure of meat wherever there was any trace of disease in the carcass. Notwithstanding their action in the matter, however, there is a general tendency both in Europe and in this country to take a more moderate view, and when the lesions are found to be localized and the flesh in good condition the carcass is not generally condemned.

With regard to milk from tuberculous animals, the experiments of Ernst and Peters, Hirschberger, and others, have demonstrated the fact that milk from tuberculous cows is dangerous, even when the udder is perfectly healthy.

The experiments of Hirschberger, which are exceedingly interesting, show that the "danger varies at different times, being present when spores from some focus of infection happened to be absorbed into the blood current and were excreted by the milk." These experiments also showed that the milk of tuberculous cows is dangerous in 55 per cent. of cases.

Bollinger, however, showed that the virulence was to a great extent lost when the milk was mixed with that of healthy cows. Negative results being obtained in one case "with dilution of 1 in 40, in another 1 in 50, in another 1 in 100." "Milk is rendered less dangerous by admixture with other milk," and while "the advancing disease in one cow increases the virulence of its milk, dilution with milk of other cows lessens the virulence."

(Annual of Medical Science, 1890, P. A 3.)

So that while there is danger in using milk from tuberculous cows, the actual danger is not so great as one might suppose if the results of laboratory experiments only were taken into consideration. The danger is principally confined to people having one cow for family use, or to children being fed with milk from a cow reserved for that purpose.

In considering the direct hereditary transmission of tuberculosis, I would call your attention to a case reported in the Journal of Comparative Pathology (March, 1892). The article goes on to say "that in the body of a man who had died of disseminated tuberculosis of the pharynx, larynx, lungs, intestine, kidneys, prostate, and rectum, the vesiculæ seminales were full of semen which were found to be swarming with tubercle bacilli. The other genito-urinary organs were healthy." (Br. Med. Jour.)

This case is peculiarly interesting and instructive, and following in the same line McFadyen reports a case of congenital tuberculosis in a calf five days old, in several of which the lymphatic glands "were enlarged to the size of a large nut, and caseous toward the centre. Some small nodules the size of a pea were seated in the liver substance itself, and these were also in a state of caseous degeneration;" "and on staining cover glass preparations there was no difficulty in discovering tubercle bacilli."

That this mode of transmission of tuberculosis is uncommon, not to say rare, is shown by the fact that up to the present time there have been observed only six cases of indubitable congenital tuberculosis in the calf, that is to say, cases in which the possibility of the disease having had an extra uterine origin was excluded, and in which the exact nature of the lesions was established by the discovery of Koch's bacilli in them. In three of these cases the lesions were discovered in the unborn fœtus, in the fourth the calf was dead born, and in the other two the animal was under fourteen days old. The writer goes on to say, "It is probably not wide of the mark to estimate tuberculosis among dairy cows at 3 per cent., and yet we know from careful statistics furnished by the large continental abattoirs that the proportion of tuberculosis among calves under one month old does not exceed 1 in 70,000."

This would tend to show that direct transmission of tuberculosis from parent to offspring is rare. As a rule, the offspring of tuberculous parents is weakly and predisposed to disease in consequence of the want of constitutional vigor to resist it. In the great majority of cases tuberculosis is not directly transmitted; there is simply a constitutional weakness and predisposition, not perhaps especially to tuberculosis, but to disease in general.

Among the many predisposing causes of tuberculosis I would class anything that lowers or tends to lower the vitality of the system, anything that decreases or tends to decrease the disease resisting powers which all healthy animals possess to a high degree ; and hereditary predisposition is probably one of the foremost of these causes, because for generations cattle have been bred for their milk supply with a total and suicidal disregard for the general health and strength of the animal.

Among the causes that have tended to produce this peculiar constitutional predisposition of dairy cattle probably the most important are confinement and want of exercise, poor ventilation and bad sanitary condition generally, with injudicious feeding and breeding.

A German journal states "that in the Canton Feirburg, in 1890, out of 14,142 housed animals there were 249 deaths, and 8.7 of these deaths were due to tuberculosis, while in the district in which the cattle were fed out of doors, the deaths from tuberculosis amounted to only 3 per cent. of the total losses—that is to say, in the districts where the animals were almost constantly housed and fed unnaturally the deaths were 2½ times greater than where the animals were kept out of doors and fed naturally."

This is only what one would expect, as it is in close, ill-ventilated barns that the bacilli would naturally collect ; and apparently no better proof of this could be found than the remarkable results obtained through improvement in ventilation in the French Cavalry Stables.

After sanitary measures were adopted cases of glanders in the cavalry horses fell from 23.32 per 1,000 in 1874-52 to 7.24 per 1,000 in 1862-66, and during the same period cases of non-specific diseases of the lungs fell from *104.7 per 1,000 to 3.59 per 1,000.* The only condition present to account for this remarkable change was increased facility for ventilation and increased cubic space. (Paper read at Vet. Con. of H. & D., 1890.)

Now we know that the average dairy barn is very imperfect in this respect. I have seen barns so close that a lantern hung up in the barn in the evening would go out before morning for want of air.

One can hardly go into any barn in the morning without feeling the hot, close smell of the cattle, so strong sometimes as to be almost overpowering, and yet the average farmer will tell you when he has a barn of this description that he has a nice, comfortable, warm barn. The hotter and closer it is the more comfortable he seems to consider it.

Drainage is another important matter that is too often entirely neglected; it is just as important to have the cow barn and yard well drained as it is to have the house and surroundings well drained.

The statistics collected by Dr. Buchanan on this subject are highly suggestive, and their importance must be my excuse for their introduction here.

In Salisbury, Eng., after the introduction of improved drainage, the annual death rate from phthisis fell from $44\frac{1}{2}$ per 10.000 to $22\frac{1}{3}$ per 10,000 between 1837-64. In Banbury the phthisis death rate fell in the same length of time from $26\frac{2}{3}$ to $15\frac{3}{5}$ per 10,000.

In the same period of time in the towns of Ely, Rugby, Worthing, Macclesfield, Leicester, Newport, the death rate fell 47 per cent., 43 per cent., 36 per cent., 51 per cent., 52 per cent., and 52 per cent. respectively, in consequence of improvement in drainage alone. And yet this is almost the last thing the farmer thinks about.

Beneath and around the barns stand piles of rotting manure. The urine soaks into and through the wooden floor and drips into the cellar beneath; while the yards and surroundings are usually a mass of decaying animal and vegetable matter.

The surface water from this mass of filth, often draining into the well, which is usually located in or near the barn yard, forming a cesspool for the collection of surface drainage.

As for good light, every school boy knows how essential it is, yet if not wanting entirely, the light is often a mere apology for that commodity. Cattle need light every whit as much as either plant or human being, and yet I could take you to day to barns that are supposed to be first class in every respect, where one needs a lantern to find out whether the pens are occupied or not.

Perhaps, however, the most important factor in predisposing the dairy cattle to tuberculosis is the injudicious management of the dairy stock themselves.

It is generally recognized in the medical profession that when a woman becomes pregnant she should not be allowed to nurse her child, otherwise both herself and her offspring will be the worse for it, both will suffer in health.

Fagge says, "In the female, childbearing seems to play an important part among the causes of phthisis; and according to

Dr. Pollock it is not so much prolonged suckling that seems to set up phthisis, but the mere fact of suckling at all. Cases associated with childbirth generally run a particularly acute course."

In referring to the foregoing Dr. Pye Smith says, "These considerations are so important in regard to life insurance that Sir Risdom Benett and myself have for several years advised the office with which we are connected to count all deaths of mothers 'in childbed' or 'after delivery' as due to phthisis, unless there is explicit evidence of previous good health." This is, possibly, an extreme view, but if it is the case, or even if it is only in part true, what must be the result when the dairy cow is not only milked when pregnant, but milked right up to the time of calving; they are fed so as to produce the greatest possible quantity of milk, and every year the dairy cow is expected to bear a calf with unfailing regularity; and when one remembers that this process is kept up, not for one generation only, but for generation after generation, the wonder is that tuberculosis is not far more common than it is, for we have here *just the very conditions that are most fitted for its development.* Constitutions weakened and vitality lowered through generations of injudicious breeding along with poor sanitary conditions, poor ventilation, poor light, and bad drainage, *what more do we want, what more could we have, even if we wanted to propagate the disease instead of controlling it?*

*Prevention.*—In considering the measures to be adopted by the authorities for the purpose of controlling the spread of tuberculosis, we must not forget that the disease is not confined to one species of animal, but it may be communicated to and by all kinds of animals, and so long as there remains one tuberculous individual (whether man or animal) there will be danger to all other animals.

But while it is not possible to eradicate it by the process of killlng off diseased animals, it is possible to control it to a great extent by improving the surroundings and the sanitary and hygienic conditions of the cattle, and by changing the present method of breeding and feeding to one more in accordance with the dictates of common sense.

In the first place the present provision of the state law ought to be thoroughly enforced; a competent veterinary surgeon should be appointed as inspector in every city and town of the commonwealth; every dairy should be licensed and periodically inspected (say monthly or bi-monthly); each cow ought to be registered and carefully examined for any trace of disease, an especial examination being made of the udders; and no cow ought to be used for dairy purposes unless it is in a generally healthy condition

And further, as suggested by Prof. Wally, "Whenever such disease is detected, power should be given to the inspector to remove the animal to a sanitarium, or other place, for further observation, or to an abattoir foı slaughter; if in the latter case it were ultimately found on autopsy that no disease existed of a nature likely to render the meat or milk harmful, compensation should be given the owner of the cow."

The barns ought also to be periodically inspected for their sanitary condition, which should include the cubic space for each animal, ventilation, drainage, light, cleanliness, water supply, etc.

In this connection I would call your attention to the granting of licenses to dairies in other countries.

In Scotland an act was passed called "The dairies, cow sheds and milk shops order of 1885," in which the police committee as local sanitary authority are empowered to make regulations *inter alia*, for prescribing and regulating the lighting, ventilating, cleansing, drainage, and water supply of dairies and cow-sheds in the occupation of persons following the trade of cow-keepers or dairy men, and the order declares that no cow-shed shall be occupied, if new, until provision is made "to the reasonable satisfaction of the local authority for the lighting and the ventilation, including air-space, etc.; and no cow-shed whatever shall continue to be occupied if, and as long as the lighting and ventilation, including air-space, are not such as are necessary and proper for the health and good condition of the cattle therein;" and in a report to the Glasgow Board of Health, Dr. Russell, the medical officer of health, recommends—

1st, "That the registration, regulation, and control of byers should be placed in control of the sanitary authority.

2d, "That in all existing byers the cubic space should be raised to 600 cubic feet; that in all new byers it should be 800 cubic feet, and that the regulations generally as to lighting, ventilation, drainage, cleansing, and water supply should be carefully revised so as to give full effect to the mind of the sanitary authority, and thereby enable them to discharge themselves of the responsibility imposed upon them by the Legislature." (Mc. F., vol., p. 96.)

Denmark and Italy are, I believe, the only countries in the Continent of Europe that pay any special attention to the sanitary condition of the dairies; Great Britain and other countries are fast beginning to realize the importance of the subject, and it would be well if some such regulation as the above, regulating the granting of licenses to dairies, were in force here.

Again in the matter of meat supply public abattoirs should be established and all private abattoirs should be abolished or licensed and made subject to inspection at all times.

France and Germany take the lead in this respect. In Berlin all meat is inspected and stamped with a government stamp before being exposed for sale.

Special officers are appointed to see that no meat is smuggled in without inspection, and all meat found exposed for sale without the government stamp is confiscated and the seller prosecuted.

"In Berlin the staff engaged in meat inspection at the central abattoir comprises (besides common workmen) 250 persons, including the director, 19 veterinary surgeons, 6 assistant veterinary surgeons; other 15 veterinary surgeons are engaged in the inspection of carcasses slaughtered and brought into the city." (Jour. Com. Path., vol. 3, p. 188.)

Now, while it might not be advisible to go the length Germany has gone in the matter, yet here we have not gone far enough. It is the duty of the government to protect the public by making meat inspection compulsory and to provide competent meat inspectors. It is much more the duty of the government to see that dairies, from which the public get their milk supply, are clean and in good order and that the sanitary conditions and surroundings are as nearly perfect as possible.

It is true that an enforcement of dairy inspection might result in hardship to certain individuals, but the public have a right to claim protection.

It may also be urged that it would be almost impossible to control the milk supply of a large city like Boston, which receives its milk supply from all the surrounding country, but it seems to me it would be a simple enough matter if the State authorities had the power to grant licenses and were to divide the State into districts, each district being in charge of an inspector, whose duty it would be to inspect all dairies in his district; no person being allowed to sell milk without a license.

And, in conclusion, until some regulations are in force here, and until greater attention is paid to the health and constitution of the dairy stock and to the cleanliness and sanitary condition of the barns and their surroundings, all efforts to control and reduce tuberculosis in our dairy cattle must result in failure.

